# ZEB1-induced LINC01559 expedites cell proliferation, migration and EMT process in gastric cancer through recruiting IGF2BP2 to stabilize ZEB1 expression

**DOI:** 10.1038/s41419-021-03571-5

**Published:** 2021-04-06

**Authors:** Huojian Shen, Hongyi Zhu, Yuanwen Chen, Zhiyong Shen, Weiqing Qiu, Changlin Qian, Jie Zhang

**Affiliations:** Renji Hospital Affiliated to Medical College of Shanghai Jiaotong University, Shanghai, 200025 China

**Keywords:** Gastric cancer, Cell biology

## Abstract

Gastric cancer (GC) is a common type of tumor that is characterized with high metastatic rate. In recent years, increasing studies have indicated that lncRNAs are involved in the regulation on cancer cell proliferation and migration. However, the functional role of long intergenic non-protein coding RNA 1559 (LINC01559) in GC is still unclear. In this study, we applied quantitative real-time polymerase chain reaction (RT-qPCR) and examined that LINC01559 expression was significantly enhanced in GC cells. Functional assays such as EdU, colony formation, JC-1 and transwell assays displayed that silencing LINC01559 inhibited cell proliferation and migration while promoted cell apoptosis in GC. Besides, western blot analysis and immunofluorescence assays examined the expression of factors related to epithelial-mesenchymal transition (EMT) and indicated that EMT process was blocked by LINC01559 knockdown in GC cells. Besides, LINC01559 silencing inhibited tumor growth in vivo. In addition, Chromatin immunoprecipitation (ChIP) assays demonstrated that zinc finger E-box binding homeobox 1 (ZEB1) served as a transcription factor to combine with LINC01559 promoter and activated the expression of LINC01559 in GC cells. In return, LINC01559 recruited insulin like growth factor 2 mRNA binding protein 2 (IGF2BP2) to stabilize ZEB1 mRNA to up-regulate ZEB1 in GC cells. In short, the findings in this research might provide a novel target for GC treatment.

## Introduction

Nowadays, gastric cancer (GC) is one of the most common cancers worldwide^1^. In East Asia, especially in Korea, Mongolia, Japan, and China, GC-associated morbidity and mortality have been shown to be relatively higher than in most western countries^2,3^. Despite advances in diagnostic techniques and improvements in chemotherapy and radiotherapy, patients with advanced or metastatic GC still present a poor prognosis^4^. One of the main causes of cancer-related mortality is tumor metastasis. Therefore, early diagnosis of GC is of significant importance for prognosis^5^.

Long non-coding RNAs (lncRNAs) are a type of discovered RNAs with the length over 200 nucleotides and without the ability of coding proteins^6,7^. LncRNAs are involved in a wide range of biological processes in various cancers, such as cell proliferation, migration and apoptosis^8^. Abnormally expressed lncRNAs can serves as oncogenes or tumor suppressors to affect the progression and metastasis of cancers^9,10^. Mechanistically, lncRNAs exert their functions majorly through the interaction with RNAs or proteins in the regulation of gene expression^11^. In recent years, a large number of studies have shown that lncRNAs play vital roles in the progression of GC, such as MEG3^12^, HOXA11-AS^13^ and LINC00337^14^. However, there are still some unknown lncRNAs whose mechanisms need to be further explored in GC.

Zinc finger E-box binding homeobox 1 (ZEB1) is a transcription factor registered by some studies. In prostate cancer cells, ZEB1 is a key promoter of tumor progression^15^. In breast cancer cells, ZEB1 induces transcriptional regulation of genes involved in the cancer progression^16^. Thus, it is of great interest and importance to identify the association between lncRNAs and ZEB1 in the progression of GC.

In addition to target and sequester specific microRNAs (miRNAs), lncRNAs can function in the progression of cancers through recruiting RNA-binding proteins (RBPs)^17^. Abundant studies have demonstrated the participation of RBPs in the maintenance of mRNA stability by lncRNAs in cancer cells. For instance, CERS6-AS1 promotes the progression and metastasis of breast cancer by recruiting with IGF2BP3 to increase the stability of CERS6 mRNA^18^. Besides, LIN28B-AS1 interacts with by IGF2BP1 to activate LIN28B expression in lung adenocarcinoma cells^19^. In our study, we also investigated whether LINC01559 could recruit certain protein partner to exert its function in GC cells.

Epithelial-to-mesenchymal transition (EMT) is a cellular process related to metastasis^20^. EMT is essential for embryonic development and has been associated with a variety of diseases. EMT-related transcription factors (Snail, Twist, and ZEB1) can induce EMT process in vitro and in vivo^21^. However, whether and how EMT is modulated by LINC01559 in GC cells remain to be further specified.

In conclusion, our study expounded that LINC01559 expression was up-regulated in GC cells. Knockdown of LINC01559 inhibited cell proliferation, migration and EMT process in GC. Moreover, ZEB1 could activate the expression of LINC01559, and LINC01559 recruited insulin like growth factor 2 mRNA binding protein 2 (IGF2BP2) to stabilize ZEB1 mRNA in GC cells. In a word, this study might be helpful to identify effective treatments for GC.

## Materials and methods

### Cell culture

Human GC cell lines (SNU-1 and AGS) were purchased from American Type Culture Collection (ATCC) cell bank (Manassas, VA); another two kinds of human GC cell lines (MKN45 and HGC-27) were gotten from the Institute of Biochemistry and Cell Biology, Chinese Academy of Sciences (Shanghai, China); the normal human gastric epithelial cell line (GES-1) was obtained from Procell Life Science & Technology Co., Ltd. (Wuhan, China). SNU-1, MKN45, HGC-27 and GES-1 cells were cultivated in RPMI-1640 medium (Invitrogen, Carlsbad, CA, USA). AGS cells was cultivated in F-12K medium (Invitrogen, Carlsbad, CA, USA). The culture media were all supplemented with 10% FBS and 1% P/S. All cells were grown in a humidified atmosphere of 5% CO_2_ at 37 °C.

### Cell transfection

Specific shRNAs targeting LINC01559 (sh/LINC01559#1/#2) or IGF2BP2 (sh/IGF2BP2#1/#2) and control group (sh/Ctrl) were synthesized by GenePharma (Shanghai, China). The pcDNA3.1 vectors from Invitrogen were utilized to construct pcDNA3.1/ZEB1, pcDNA3.1/MAFK, pcDNA3.1/JUND, pcDNA3.1/JUN, pcDNA3.1/ESR1 and pcDNA3.1/RFX5, with the empty vector as the negative control. Lipofectamine 3000 (Invitrogen) was used to execute transfection experiments for 48 h.

### Quantitative real-time polymerase chain reaction (RT-qPCR)

Total RNA was extracted from GC cells using the TRIzol reagent according to manufacturer’s requirements (Thermo Fisher Scientific), and then cDNA was obtained after reverse transcription. RT-qPCR was performed with SYBR Green Master Mixture (Takara, Dalian, China). GAPDH was used as the control. Relative gene expression levels were calculated using the 2^−ΔΔCt^ method. The experiment was repeated for three times.

### 5-ethynyl-2’-deoxyuridine (EdU) assay

After transfection, GC cells were seeded onto 96-well plates. The EdU (5-ethynyl-20-deoxyuridine) Apollo-567 Kit (RIBOBIO, Shanghai, China) was used to quantify the proliferation ability of indicated GC cells. EdU and DAPI dyes were exploited to treat GC cells. A fluorescent microscope was used for cell visualization (Olympus, Tokyo, Japan). The experiment was repeated for three times.

### Colony formation assay

GC cells were seeded into 6-well plate at the density of 500 cells per well. After 2 weeks, colonies were fixed with 4% paraformaldehyde and stained with 0.1% crystal violet (DINGGUO) and then counted manually. The experiment was repeated for three times.

### Flow cytometry analysis

The apoptosis rate of SNU-1 and AGS cells was analyzed with propidium iodide (PI)/Annexin V Cell Apoptosis Kit (Invitrogen, V13245) in reference to manufacturer’s suggestions. In short, after 48 h transfection, SNU-1 and AGS cells were stained by FITC-Annexin V and PI. Finally, cell apoptosis rate was examined by flow cytometry (FACSCantoII, 338960; BD Biosciences, San Jose, CA, USA). The experiment was repeated for three times.

### JC-1 assay

Cells were incubated with 500 μl JC-1 staining working solution for 20 min at 37 °C (Beyotime, China). The fluorescence labeled cells were washed using phosphate buffered saline (PBS) and analyzed by an EnSpire Reader. The data and images were processed by GraphPad Prism 6.0 statistical software. The experiment was repeated for three times.

### Transwell assay

GC cells (1 × 10^5^) in serum-free medium were seeded on the upper transwell chamber (BD Biosciences). The complete culture medium (with 10% FBS) was added to the lower chamber. After 24 h, the migrated cells on the lower surface were fixed and stained. Five repeated views in each condition were used to calculate the number of migrated cells. The experiment was repeated for three times.

### Western blot

Total cellular lysates were resolved by RIPA lysis buffer and then proteins were separated via SDS-PAGE (10%) gels and transferred to a PVDF membrane. The latter was incubated with blocking buffer and followed by further processing with primary antibodies against GAPDH (1/10000, ab181602), E-cadherin (1:2000-1:8000, 60335-1-Ig), N-cadherin (1 µg/ml, ab18203), Slug (1:1000, 9585S), Twist (1/5000, ab187008), ZEB1 (1:500-1:2000, 21544-1-AP), IGF2BP2 (1:1000, 14672S), MAFK (1/4000-1/8000, ab50322), JUND (1:1000, 5000S), JUN (1:1000-1:8000, 66313-1-Ig), ESR1 (1:500-1:2000, 21244-1-AP) and RFX5 (1/500-1/5000, ab9255) as well as secondary antibodies. ECL method was applied to detect the immuno-reactive bands. The experiment was repeated for three times.

### Immunofluorescence analysis

SNU-1 and AGS cells were fixed with 4% paraformaldehyde, followed by the incubation with primary antibodies against E-cadherin (1:2000-1:8000, 60335-1-Ig) and N-cadherin (1 µg/ml, ab18203) overnight at 4 °C. Then cells were hatched with Cy3-conjugated secondary antibody for 30 min at room temperature. The nuclei were re-stained by DAPI for 3 min. The images were gotten via a fluorescence microscopy (Nikon Corporation) and qualified by Image-Pro Plus software. The experiment was repeated for three times.

### Tumor xenograft assay

Four-week-old female athymic nude mice were purchased from Renji Hospital Affiliated to Medical College of Shanghai Jiaotong University. This study was approved by the Ethic Committee of Renji Hospital Affiliated to Medical College of Shanghai Jiaotong University. 1 × 10^7^ cells transfected with sh/Ctrl or sh/LINC01559#1 were injected subcutaneously into the randomly divided two groups of nude mice. The tumor volume was tested every four days and tumor growth lasted for 4 weeks. After that, the mice were mercy killing and their tumors were taken out. The tumor weight was calculated and the tumors were collected for following analysis.

### Immunohistochemical (IHC) assay

The collected tumor tissues from xenograft assay were dehydrated after fixing in 4% paraformaldehyde, and then embedded in paraffin and cut into sections of 4 μm thick. Then the sections were utilized for IHC assay by use of specific antibody to Ki-67, PCNA, E-cadherin or N-cadherin (Abcam, Cambridge, MA). The experiment was repeated for three times.

### Chromatin immunoprecipitation (ChIP) assay

ChIP analysis was carried out using the Millipore ChIP Assay Kit (Millipore, MA, USA) based on the manufacturer’s instructions. SNU-1 and AGS cells were cross-linked with formaldehyde and sonicated into 200–1000 bp. DNA samples were precipitated with anti-IgG or anti-ZEB1 antibody upon 30 μl of magnetic beads for 6 h. Then, the precipitated chromatin DNA was collected, extracted and purified for RT-qPCR analysis. The experiment was repeated for three times.

### Luciferase reporter assay

The sequence of wild-type or indicated site-mutated LINC01559 promoter (WT, Site 1-MUT, Site 2-MUT, Site 3-MUT, Site 4-MUT, Site 5-MUT, and MUT) were synthesized and cloned into pGL3 vector to construct corresponding reporter plasmids. Then, indicated recombinant reporter was co-transfected with pcDNA3.1/ZEB1 or pcDNA3.1 into SNU-1 and AGS cells. After 48 h, the luciferase activity of each group was detected using Dual-Luciferase Reporter Assay System (Promega). The experiment was repeated for three times.

### Fluorescence in situ Hybridization (FISH) assay

Fluorescently-labeled LINC01559 probe was constructed by RiboBio. The FISH Kit (RiboBio) was used for the detection of LINC01559 signals. DAPI was utilized to re-stain the nuclei of SNU-1 and AGS cells. Images were acquired through a confocal microscope (Olympus, Tokyo, Japan). The experiment was repeated for three times.

### Subcellular fractionation

Nuclear or cytoplasmic RNA in SNU-1 and AGS cells was separated and purified with the application of a Cytoplasmic and Nuclear RNA Purification Kit (Norgen Biotek, Thorold, Canada). The percentage of LINC01559 in nuclear or cytoplasm fractions was examined with RT-qPCR. GAPDH and U6 were the control for cytoplasmic or nuclear RNA, respectively. The experiment was repeated for three times.

### Actinomycin D experiment

SNU-1 and AGS cells were seeded in 24-well plates (5 × 10^4^/well). After transfection, cells were exposed to Actinomycin D (2 μg/ml, Abcam, ab141058) for 0 h, 4 h and 8 h. Then the relative mRNA level of ZEB1 was examined by RT-qPCR and normalized to the values measured in the 0 h group (mock treatment). The experiment was repeated for three times.

### RNA pull-down assay

Biotin-labeled full-length and antisense LINC01559 or ZEB1 sequences were obtained by the use of Transcript Aid T7 High Yield Transcription Kit (Thermo Scientific). Then the MEGAclearTM Kit (Thermo Scientific) was applied to recycle the sequences in line with manufacturer’s advice. The sequences were incubated with cell lysates for 4 h at 4 °C, and then the biotin-labeled RNAs and their binding protein partner were pulled down by streptavidin magnetic beads (Thermo, USA) at 4 °C overnight. The proteins were separated by electrophoresis and visualized using the Coomassie Blue Staining Kit (Beyotime, China). The different bands between sense and antisense LINC01559 were identified using mass spectrometry and determined through western blot analysis. The experiment was repeated for three times.

### RNA immunoprecipitation (RIP) assays

RIP assay was conducted to study whether LINC01559 or ZEB1 interacted with IGF2BP2 by using a Magna RIP RNA-binding protein immunoprecipitation kit (Millipore, Billerica, MA, USA). Cells were lysed with RIP buffer, and then the cell extract was mixed with agarose beads carrying anti-IGF2BP2 or anti-lgG antibody. The enrichment of co-precipitated RNAs was detected using RT-qPCR. The experiment was repeated for three times.

### Statistical analysis

Statistic data of each group were determined using GraphPad 6.0. Data were expressed as mean ± standard deviation (SD). Intergroup difference was evaluated using Student’s *t* test, and multivariate statistical analysis was assessed using Analysis of variance (ANOVA). *P* < 0.05 was considered with statistical significance. All experiments were repeated for three times.

## Results

### Knockdown of LINC01559 suppresses cell proliferation and promotes cell apoptosis in GC

**Fig. 1:**
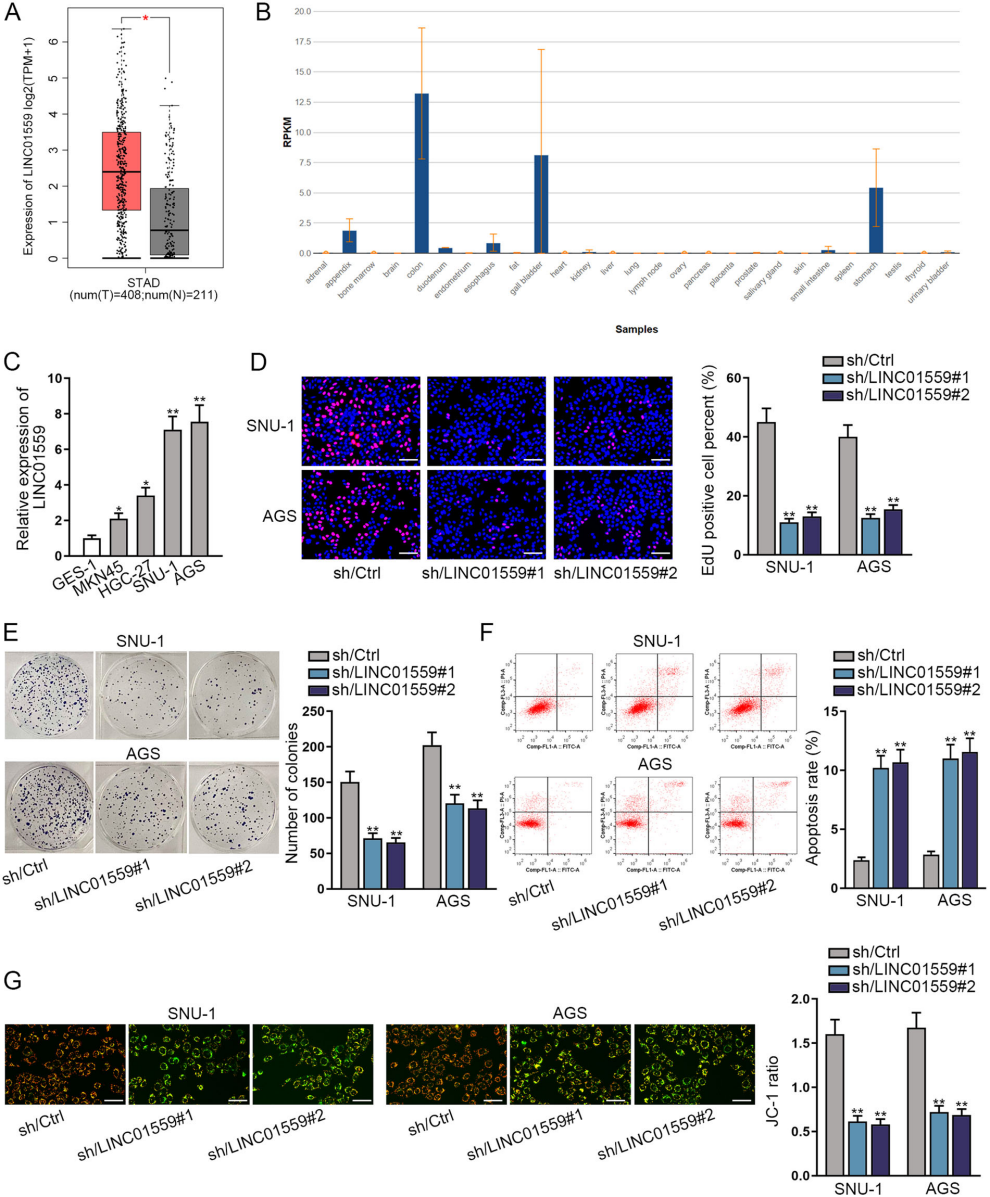
Knockdown of LINC01559 suppresses the proliferation and promotes the apoptosis in GC cells. **A** GEPIA2 analyzed LINC01559 expression in 408 STAD tissues and 211 normal gastric tissues. **B** NCBI data showed LINC01559 expression profile in different normal tissues. **C** The relative expression level of LINC01559 in four GC cell lines (MKN45, HGC-27, SNU-1 and AGS) and GES-1 cells was examined by RT-qPCR analysis. **D, E** EdU and colony formation experiments tested the proliferation capacity of SNU-1 and AGS cells with or without LINC01559 silence. **F, G**. Flow cytometry analysis and JC-1 assays detected the apoptosis in SNU-1 and AGS cells before or after LINC01559 silence. *P < 0.05, **P < 0.01.

First, we screened the data from GEPIA2 (http://gepia2.cancer-pku.cn/#analysis) and found that LINC01559 expression was higher in 408 stomach adenocarcinoma (STAD) tissues compared with that in 211 normal gastric specimens (Fig. 1A). Simultaneously, data from NCBI (https://www.ncbi.nlm.nih.gov/) indicated that LINC01559 expression was low in normal human stomach tissues (Fig. 1B), which implied that LINC01559 was aberrantly expressed in cancerous situation. Then, the expression level of LINC01559 in four GC cell lines (MKN45, HGC-27, SNU-1 and AGS) relative to the human normal gastric epithelial cell line (GES-1) was detected by RT-qPCR analysis. Results manifested that the expression level of LINC01559 in four GC cell lines was significantly up-regulated compared with that in GES-1 cells, and its expression in SNU-1 and AGS cells was relatively highest (Fig. 1C). Therefore, these two cancer cell lines were screened out for follow-up experiments. We stably knocked down the expression of LINC01559 in SNU-1 and AGS cells, and confirmed the knockdown efficiency of LINC01559 by sh/LINC01559#1 and sh/LINC01559#2 using RT-qPCR (Fig. S1A). Then the data from EdU and colony formation experiments showed that after silencing LINC01559, the proliferation capacity of two cancer cells was significantly reduced (Fig. 1D, E). In contrast, flow cytometry analyzed that in the absence of LINC01559, the apoptosis rate increased by approximately three times (Fig. 1F). The data of following JC-1 assay further verified that silencing of LINC01559 had a promoting effect on the apoptosis of GC cells (Fig. 1G). In short, knockdown of LINC01559 restrains the growth of GC cells.

### LINC01559 silencing inhibits GC cell migration and EMT in vitro and hinders tumor growth in vivo

**Fig. 2:**
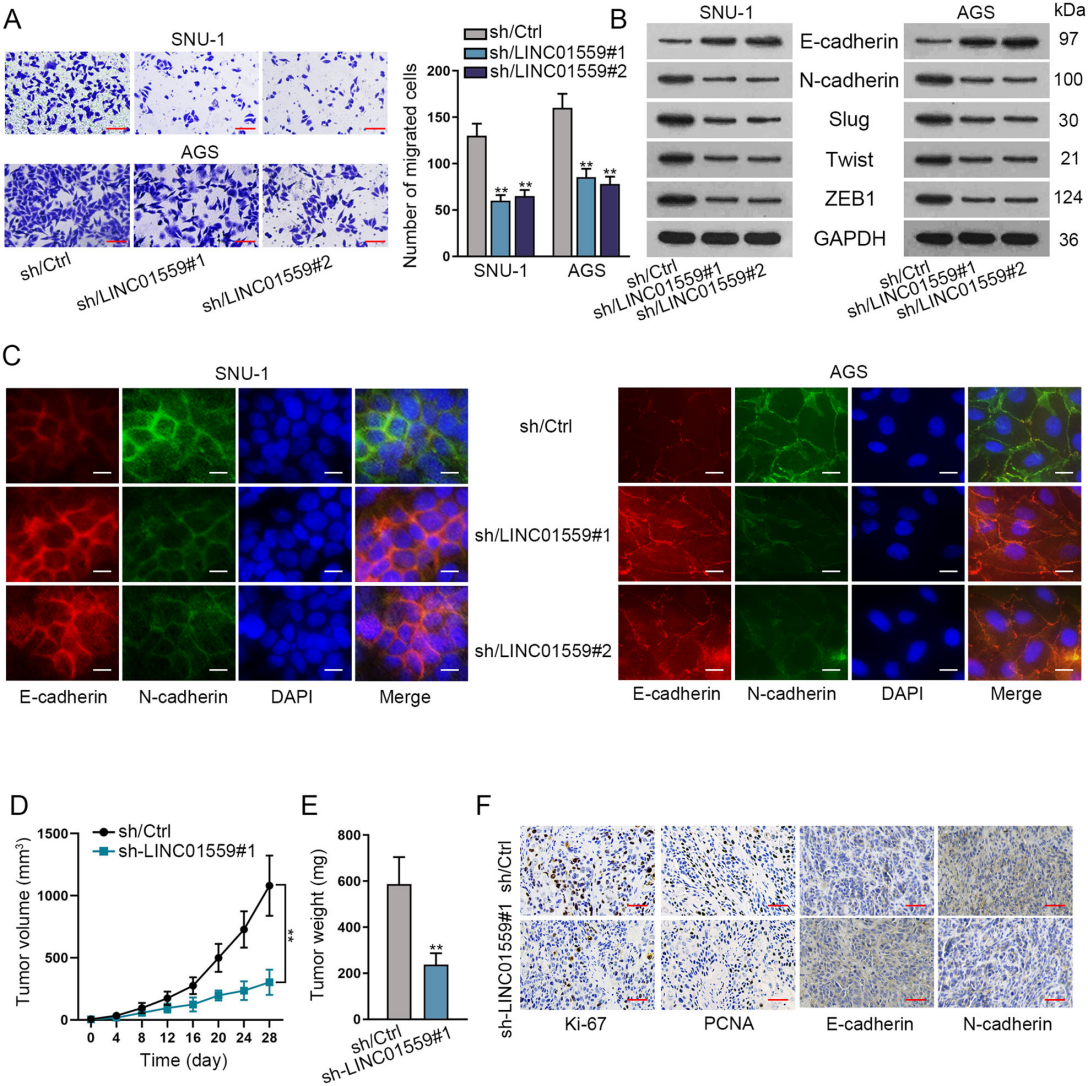
LINC01559 silencing inhibits the in vitro migration and EMT process and in vivo tumor growth of GC cells. **A**. Transwell experiment assessed the migration ability of GC cells after the interference of LINC01559. **B**, **C** Western blot and immunofluorescence experiments examined the impact of the interference of LINC01559 on the EMT process in GC cells. **D**, **E** The growth rate and weight of in vivo tumors originated from GC cells with or without LINC01559 knockdown. **F** Immunohistochemical experiment analyzed the staining of Ki-67, PCNA, E-cadherin and N-cadherin in above tumors. **P < 0.01.

Subsequently, we further investigated whether LINC01559 affected the behaviors of GC cells regarding metastasis, such as cell migration and EMT. Hence, we conducted transwell experiments and uncovered that the migration ability of GC cells was decreased after the interference of LINC01559 (Fig. 2A). EMT is a biological process in which epithelial-derived malignant cells transform into mesenchymal cells, and this process is related to cell migration and invasion^22^. Thus western blot analysis and immunofluorescence experiments were conducted to further verify whether LINC01559 affected EMT process in GC cells. The data from western blot analysis indicated that the expression of E-cadherin (epithelial marker) was increased while the levels of N-cadherin (mesenchymal marker) and EMT-related proteins including Slug, Twist and ZEB1 were decreased after the depletion of LINC01559 in GC cells (Figs. 2B and S1B). Consistently, the results from immunofluorescence experiments further validated that LINC01559 deficiency led to increased E-cadherin expression and reduced N-cadherin expression in both the two GC cells (Fig. 2C). Furthermore, we also carried out in vivo investigations to further test the role of LINC01559 in GC. As expected, silencing of LINC01559 effectively interfered the tumor growth, resulting in lighter tumor weight (Fig. 2D, E). In addition, the outcomes of immunohistochemical experiment displayed that the positivity of oncogenic Ki-67, PCNA and N-cadherin was declined while that of tumor-suppressive E-cadherin was enhanced in tumors with the interference of LINC01559 (Fig. 2F). In sum, silencing of LINC01559 inhibits the metastasis and growth of GC cells.

### ZEB1 activates LINC01559 expression in GC cells

**Fig. 3:**
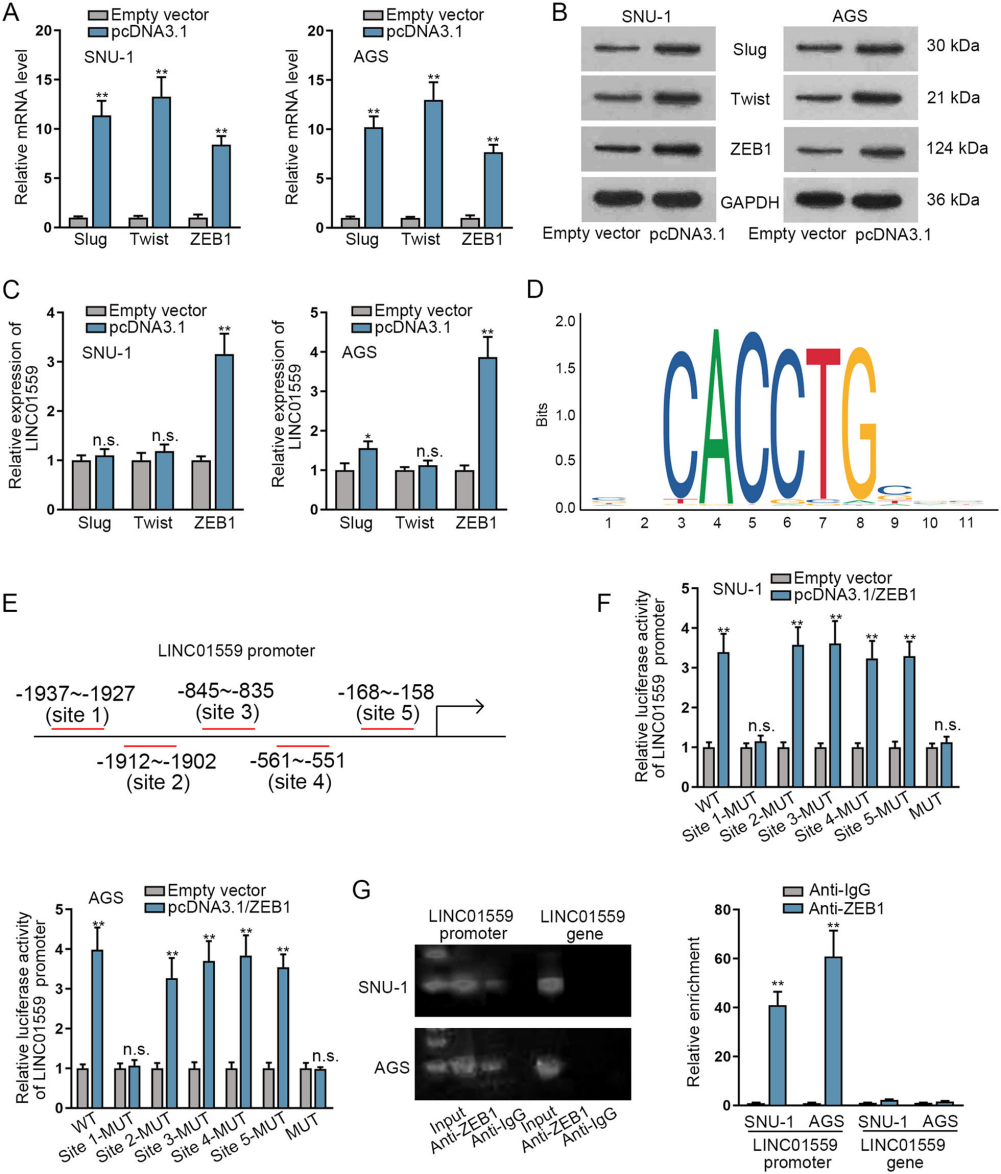
ZEB1 induces the transcriptional activation of LINC01559 in GC cells. **A**, **B** RT-qPCR and western blot examined the relative mRNA level and protein level of Slug, Twist and ZEB1 in SNU-1 and AGS cells transfected with pcDNA3.1 targeting them. **C** RT-qPCR was used to detect the expression of LINC01559 in SNU-1 and AGS cells transfected with pcDNA3.1 targeting Slug, Twist and ZEB1. **D**, **E** JASPAR website predicted the DNA binding motif for ZEB1 and the five binding sites of ZEB1 in LINC01559 promoter region. **F** Luciferase reporter experiments were conducted to detect the luciferase activity of indicated LINC01559 promoter in SNU-1 and AGS cells with or without ZEB1 overexpression. **G** ChIP assay further verified the enrichment of LINC01559 promoter in anti-ZEB1 groups. LINC01559 gene severed as the negative control of LINC01559 promoter. **P < 0.01, n.s. meant no significance.

Considering the up-regulation of LINC01559 in GC cells and its induction during EMT, we next investigated the transcriptional regulation of LINC01559. Here, we overexpressed several transcription factors in GC cells, and RT-qPCR as well as western blot confirmed the overexpression effectiveness of them in two GC cells (Figs. 3A, B and S1C, D). Thereafter, RT-qPCR data revealed that the expression level of LINC01559 was significantly up-regulated only by ZEB1 overexpression, while no obvious changes affected by the up-regulation of other transcription factors (Figs. 3C and S1E), suggesting that ZEB1 could activate LINC01559 expression. Then JASPAR website (http://jaspar.genereg.net/) provided the binding motif of ZEB1 and also predicted five binding sites for ZEB1 in LINC01559 promoter (Fig. 3D, E). To further verify the specific interacting location between them, luciferase reporter experiments were conducted. The results displayed that ZEB1 overexpression led to an increase in the luciferase activity of LINC01559 promoter that was wild-type or with site 2/3/4/5 mutations in SNU-1 and AGS cells, while no alteration was observed in that of LINC01559 promoter with Site 1 or all five sites mutated (Fig. 3F). This indicated that ZEB1 bound to Site 1 in the LINC01559 promoter. Further, ChIP assay data disclosed that the fragment of LINC01559 promoter region rather than LINC01559 gene region was highly enriched in anti-ZEB1 groups (Fig. 3G), proving the specific combination between LINC01559 promoter and ZEB1 in GC cells. All these results showed that ZEB1 activates the transcription of LINC01559 in GC cells.

### LINC01559 combines with IGF2BP2 in GC cells

**Fig. 4:**
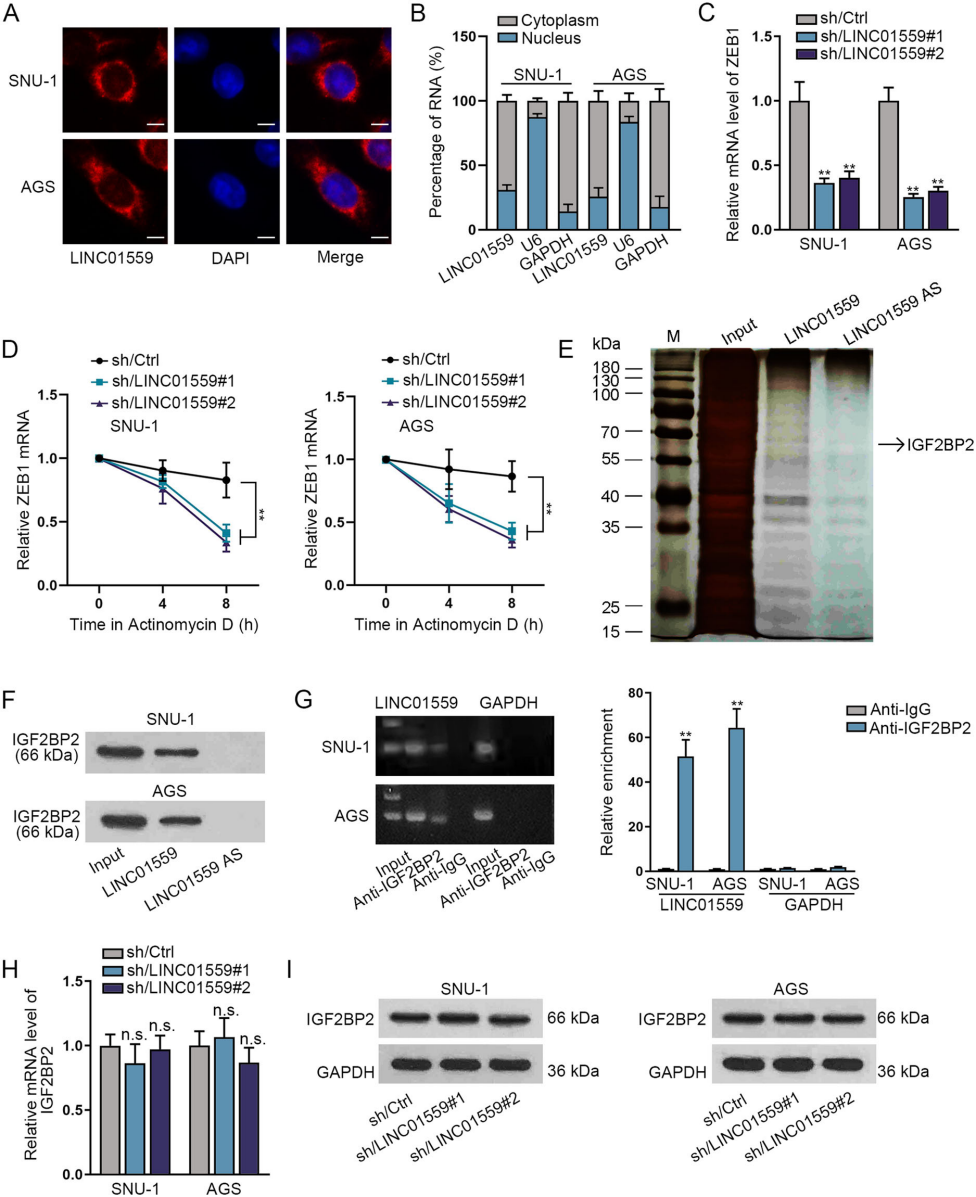
LINC01559 combines with IGF2BP2 in GC cells. **A, B** LINC01559 mainly existed in the cytoplasm of GC cells, as detected through FISH experiment and subcellular fractionation experiment**. C** RT-qPCR detected the mRNA level of ZEB1 after LINC01559 silencing in SNU-1 and AGS cells. **D** Actinomycin D treatment followed by RT-qPCR analyzed the stability of ZEB1 in SNU-1 and AGS cells with or without LINC01559 silencing. **E** Electrophoretogram revealed that IGF2BP2 was a potential protein combined with LINC01559 using RNA pull-down assay and mass spectrometry analysis. **F** The enrichment of IGF2BP2 in LINC01559 groups of SNU-1 and AGS cells was validated through RNA pull-down assay. **G** RIP experiment found the content of LINC01559 in the precipitate of anti-IGF2BP2, with GAPDH mRNA as the negative control of LINC01559. **H**, **I** RT-qPCR and western blot were conducted to detect the effect of LINC01559 silencing on IGF2BP2 mRNA level and protein level. **P < 0.01, n.s. meant no significance.

After confirming the promoting role of LINC01559 in GC, we further investigated the regulatory mechanism of LINC01559. Before that, we found that LINC01559 mainly existed in the cytoplasm based on the data from FISH experiment and subcellular fractionation experiment (Fig. 4A, B), suggesting that LINC01559 might exert effects in GC cells through post-transcriptional regulation. Interestingly, we detected that the level of ZEB1 was in return decreased by LINC01559 silencing in SNU-1 and AGS cells (Fig. 4C). According to the broken line diagram obtained by PCR and the analysis results of actinomycin D, it could be clearly seen that ZEB1 expression was declined faster after LINC01559 interference (Fig. 4D), reflecting the stability of ZEB1 mRNA was regulated by LINC01559. To investigate how LINC01559 could stabilize ZEB1 mRNA in GC cells, we performed RNA pull-down assay to identify the protein partners of LINC01559. As a result, we focused on a specific band appeared on the electrophoretic gel at about 66 kDa in LINC01559-pulled down products and then found that it was IGF2BP2 protein interacting with LINC01559 after analysis via mass spectrometry (Fig. 4E). We then observed the high enrichment of IGF2BP2 protein in LINC01559 groups through RNA pull-down assay plus an independent immunoblot (Fig. 4F). In addition, according to RIP experimental results, we found that the strong existence of LINC01559 in the precipitate of anti-IGF2BP2 in comparison to the control group (Fig. 4G). Subsequently, RT-qPCR and western blot were conducted to detect whether LINC01559 could regulate IGF2BP2 expression in GC cells. The experimental results showed that the mRNA level and protein level of IGF2BP2 exhibited no significant difference after the silence of LINC01559 (Fig. 4H, I). Totally, LINC01559 can bind with IGF2BP2 in GC cells.

### LINC01559 recruits IGF2BP2 to stabilize ZEB1 mRNA in GC cells

**Fig. 5:**
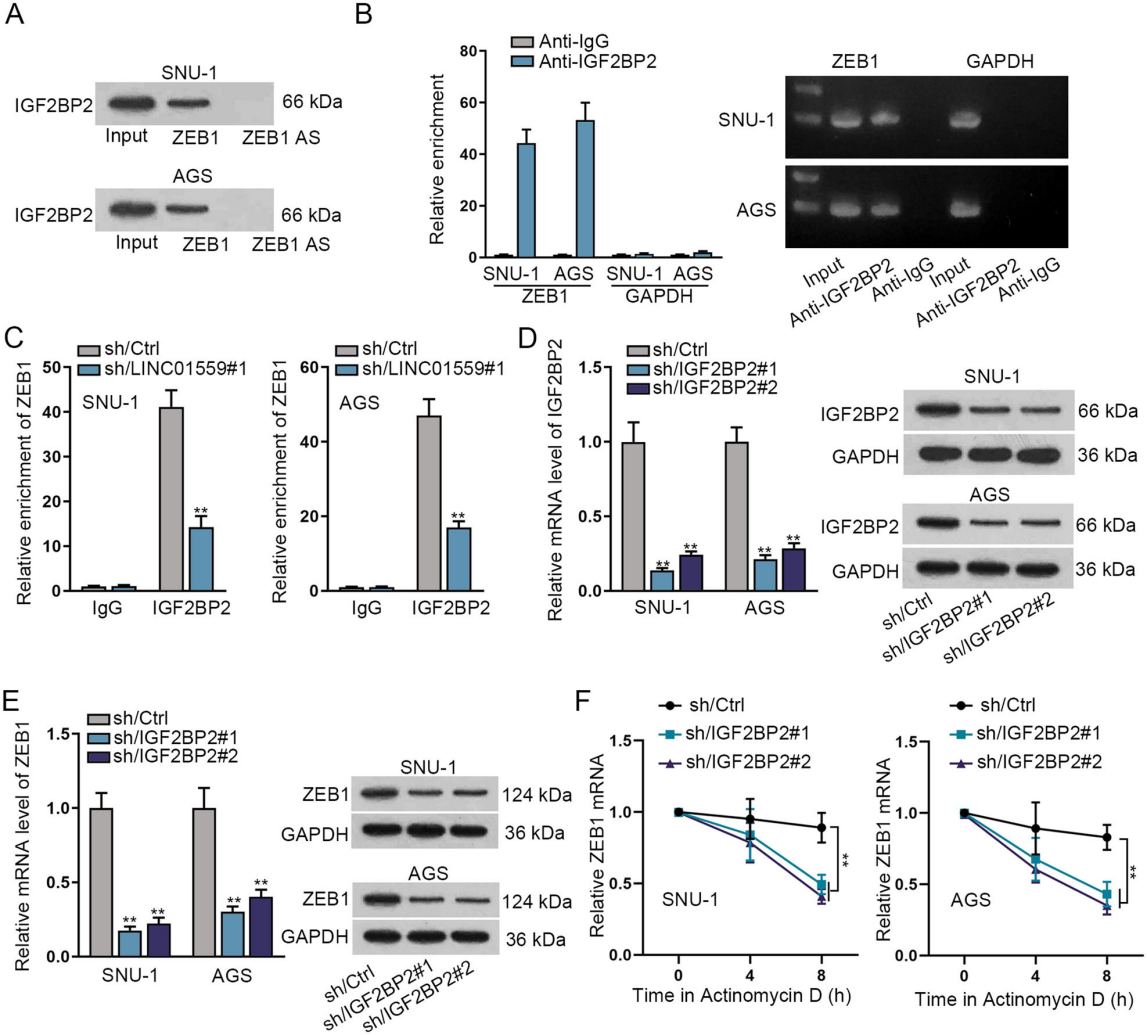
LINC01559 recruits IGF2BP2 to stabilize ZEB1 mRNA in GC cells. **A** RNA pull-down experiment was conducted to detect the enrichment of IGF2BP2 in ZEB1 groups. **B** RIP assay showed the enrichment of ZEB1 in anti-IGF2BP2 groups, with GAPDH as the negative control of ZEB1 mRNA. **C** RIP assay detected the changes in the enrichment of ZEB1 by anti-IGF2BP2 in GC cells with LINC01559 silencing. **D**, **E** RT-qPCR and western blot analyses assessed the mRNA level and protein level of IGF2BP2 and ZEB1 in GC cells transfected with sh/Ctrl or sh/IGF2BP2#1/2. **F** The stability of ZEB1 mRNA in GC cells with or without IGF2BP2 interference was assessed by RT-qPCR after treating with Actinomycin D. **P < 0.01.

Then we investigated the interaction between IGF2BP2 and ZEB1 mRNA in GC cells. The data of RNA pull-down assay presented that IGF2BP2 was obviously pulled down by ZEB1 but not by antisense ZEB1 (Fig. 5A). Then, RIP assay showed that ZEB1 was effectively enriched in anti-IGF2BP2 groups (Fig. 5B). Notably, the enrichment of ZEB1 in IGF2BP2 groups was dramatically decreased by LINC01559 silencing (Fig. 5C). To determine whether IGF2BP2 could regulate ZEB1, we stably silenced IGF2BP2 expression in GC cells (Fig. 5D). It was found that both the mRNA level and protein level of ZEB1 were obviously declined after the intervene of IGF2BP2 (Fig. 5E). Moreover, the stability of ZEB1 mRNA in GC cells was reduced in response to the interference of IGF2BP2 (Fig. 5F). Therefore, we concluded that LINC01559 recruits IGF2BP2 to stabilize ZEB1 mRNA in GC cells.

### LINC01559 promotes GC cell proliferation, migration and EMT process by up-regulating ZEB1 expression

**Fig. 6:**
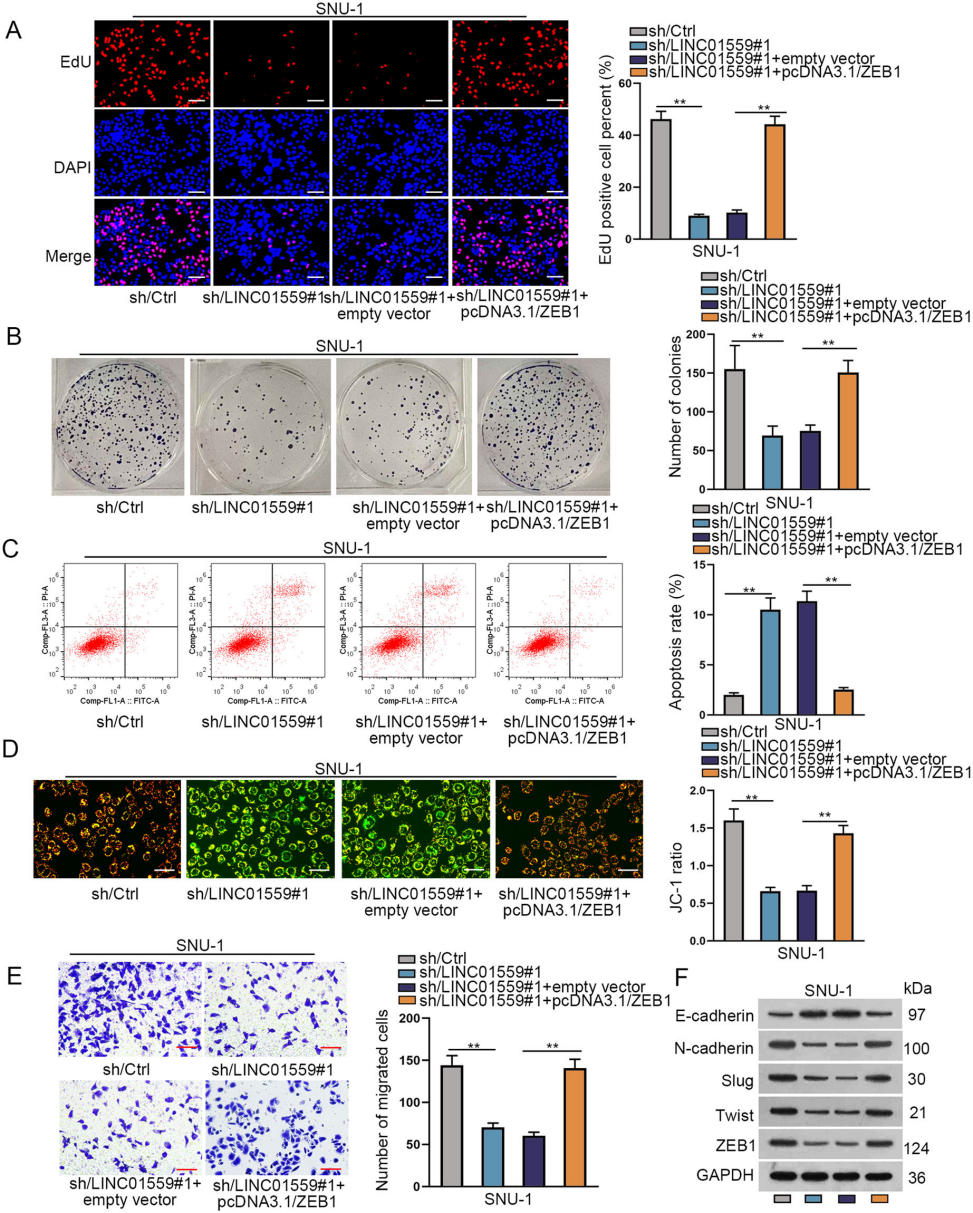
LINC01559 promotes cell proliferation, migration and EMT process by targeting ZEB1. Rescue experiments were conducted in SNU-1 cells transfected with sh/Ctrl, sh/LINC01559#1, sh/LINC01559#1 + empty vector or sh/LINC01559#1 + pcDNA3.1/ZEB1. **A**, **B** EdU assay and colony formation assay estimated the proliferation ability of SNU-1 cells in these four groups. **C**, **D** Flow cytometry analysis and JC-1 experiment analyzed the apoptotic rate of GC cells in these four groups. **E** Transwell assay determined the migration ability of GC cells in these four groups. **F** Western blot experiments examined the expression of E-cadherin, N-cadherin, Slug, Twist and ZEB1 in these four groups. **P < 0.01.

To further determine whether LINC01559 functioned in GC depending on ZEB1, rescue experiments were carried out. The results of EdU assay and colony formation assay showed that the proliferation of GC cells was significantly weakened after LINC01559 silencing, while such weakening impact was fully counteracted by the co-transfection of pcDNA3.1/ZEB1 (Figs. 6A, B and S2A, B). The data from following flow cytometry analysis and JC-1 experiments showed that the increased apoptotic rate of GC cells induced by LINC01559 silencing could be totally abolished after ZEB1 up-regulation (Figs. 6C, D and S2C, D). Moreover, the results from transwell assays revealed that the lessened number of migrated cells owing to LINC01559 knockdown could be completely rescued by ZEB1 overexpression (Figs. 6E and S2E). Finally, western blot analysis showed that the influence of LINC01559 silencing on the expression of E-cadherin, N-cadherin, Slug, Twist and ZEB1, was thoroughly offset under ZEB1 elevation (Figs. 6F and S2F, G). Together, LINC01559 contributes to GC cell proliferation, migration and EMT by up-regulating ZEB1.

### Concept map illustrates the functional role of LINC01559 in GC cells

**Fig. 7:**
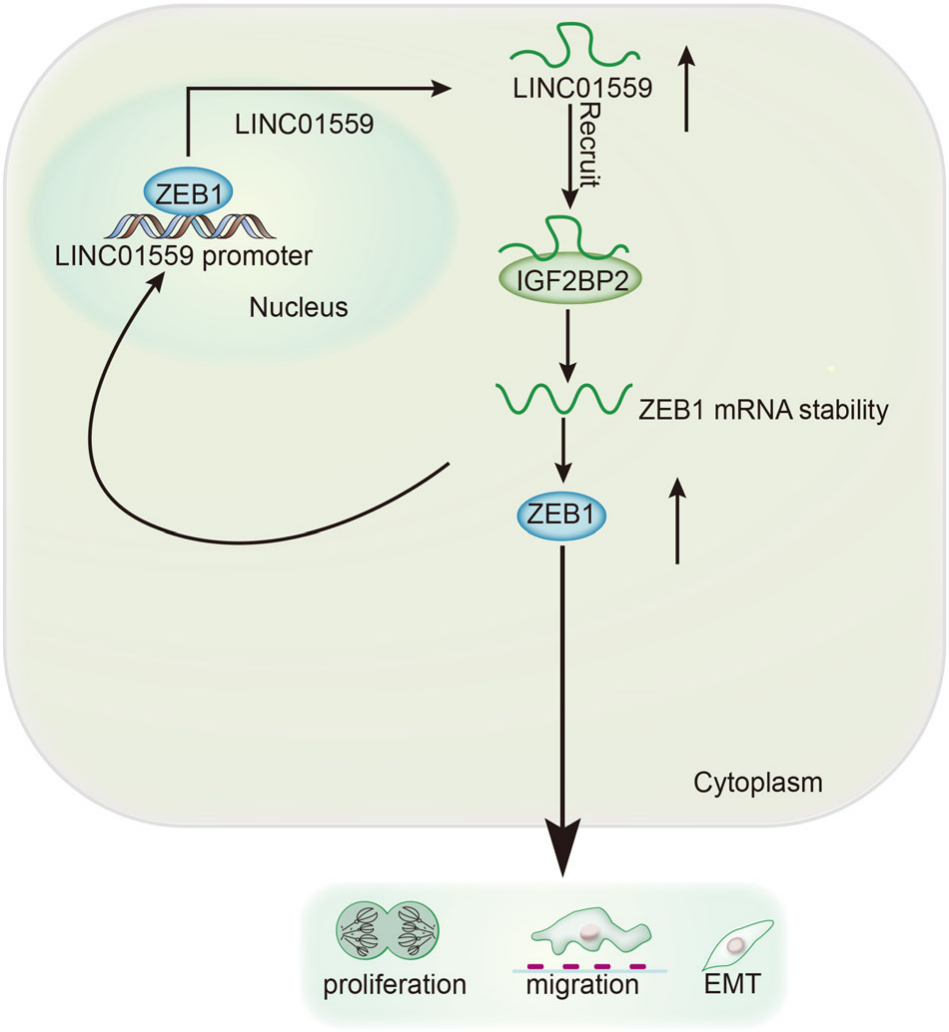
Concept map illustrates the function and regulatory mechanism of LINC01559 in GC cells.

ZEB1-induced LINC01559 facilitates cell proliferation, migration and EMT process in GC through recruiting IGF2BP2 to stabilize ZEB1 expression (Fig. 7).

## Discussion

GC is known as being metastatic and invasive^23^. Currently, the therapeutic methods for metastatic GC are very limited, and a lot of efforts have been made to develop new targeted therapies^24^. In this regard, some efforts have been put on the investigation of the relationships between lncRNAs and GC. Fu et al. have proposed that NEAT1 regulates cell proliferation, migration and invasion in GC^25^. Besides, up-regulation of Sox2ot serves as a poor prognostic marker in GC^26^. Moreover, LINC02163 contributes to GC cell growth and EMT by targeting miR-593-3p to regulate FOXK1 expression^27^. However, the role of LINC01559 in GC is rarely studied in current literatures. In this study, we found the expression level of LINC01559 was significantly elevated in GC cells. Moreover, we conducted loss-of function assays and found that silencing of LINC01559 obviously inhibited the growth and migration of GC cells, which suggested that LINC01559 could play a tumor promoting role in GC progression.

It has been reported that ZEB1 is a transcription factor related to the EMT process. Wu et al. have discovered that CASC15 affects cell proliferation, migration and EMT through targeting CDKN1A and ZEB1 in GC^28^. Besides, silencing of FoxM1 leads to the suppression of EMT in GC cells via regulating ZEB1^29^. Consistent with these reports, our study confirmed that LINC01559 could regulate the stability of ZEB1 mRNA and impede the EMT process. Notably, our study also found that ZEB1 served as a transcription factor binding to LINC01559 promoter to activate the expression of LINC01559 in GC cells. As reported previously, hypoxia-induced lncRNA-BX111 acts as an oncogene in pancreatic cancer through regulating ZEB1 transcription^30^. ZEB1-induced ZEB1-AS1 functions as a tumor-promotor in the progression of non-small cell lung cancer^31^. SOX8 activated by ZEB1 serves as a prognostic factor and development-facilitator in triple-negative breast cancer^32^. All these discoveries are in line with the finding in our study, which provide the basis of the promoting role of LINC01559 in GC cells.

Recent findings have suggested that RBPs are crucial players in post-transcriptional events. Owing to the binding versatility of their RNA-binding domains with structural flexibility, RBPs can modulate the metabolism of a large member of transcripts associated with cancer development^17^. Beside, lncRNAs have been identified to participate in the post-transcriptional control of genes through interacting with RBPs^33^. Herein, we discovered that LINC01559 mainly distributed in the cytoplasm of GC cells, and therefore we deduced that LINC01559 could have post-transcriptional regulations on genes in GC by interacting with certain RBP.

IGF2BP2 is a type of RBP that plays an important role in tumor development^34^. It has been reported that HOTAIR silencing impedes the invasion and proliferation of human colon cancer cells through regulating IGF2BP2^35^. MiR-216b is implicated in the pathogenesis and progression of hepatocellular carcinoma through regulating IGF2BP2 and HBX^36^. IGF2BP2, as well as KCNQ1 and GCKR, is associated with chemotherapeutic response and survival in patients with metastatic GC^37^. Similarly, our study screened that IGF2BP2 was certain protein partner of LINC01559. More importantly, we found that IGF2BP2 could combine with ZEB1 and regulate the stability of ZEB1 mRNA. Finally, we carried out a series of rescue experiments and verified that LINC01559 could promote GC cell proliferation, migration and EMT through recruiting IGF2BP2 to stabilize ZEB1 mRNA. As there was still a small proportion of LINC01559 in the nucleus of GC cells, we also wondered its potential role in this part. Reports have recognized that lncRNAs control gene expression via epigenetic or transcriptional regulation in the nucleus^38^. For example, HOTAIR can induce gene silencing through its interplay with PRC2^39^. Importantly, a recent study has also verified that LINC01559 can recruit E2H2 to PTEN promoter to activate PI3K/AKT pathway in GC cells^40^. This finding complements the shortcomings of our present work to some extent.

In summary, our study elucidated the oncogenic role of LINC01559 in GC cells and also figured out the LINC01559/IGF2BP2/ZEB1 axis in GC development. Our findings in present study might recommend LINC01559 as a potential therapeutic target for GC.

## Supplementary information

supplemental figure legends

Figure S1

Figure S2
