# Supplementary material for: ZEB1-induced LINC01559 expedites cell proliferation, migration and EMT process in gastric cancer through recruiting IGF2BP2 to stabilize ZEB1 expression
Source: Cell Death Dis. 2021 Apr 6;12(4):349. doi: 10.1038/s41419-021-03571-5 (PMC8024305; doi:10.1038/s41419-021-03571-5)
Supplement: Supplementary file 1 — supplemental figure legends [file 41419_2021_3571_MOESM1_ESM.docx]

***Figure S1. The expression of LINC01559 and some transcription factors was examined in GC cells.*** A. RT-qPCR examined the expression of LINC01559 in SNU-1 and AGS cells transfected with shRNAs targeting LINC01559. B. The quantification bar graphs of western blot bands of Figure 2B. C-D. RT-qPCR and western blot detected the overexpression efficiencies of MAFK, JUND, JUN, ESR1 and RFX5 in SNU-1 and AGS cells by corresponding recombinant pcDNA3.1 plasmids. E. RT-qPCR detected the expression of LINC01559 in SNU-1 and AGS cells after the overexpression of indicated transcription factors. ^**^P<0.01, n.s. meant no significance.

***Figure S2. LINC01559 promotes GC cell proliferation, migration and EMT process by up-regulating ZEB1.*** Rescue experiments were conducted in AGS cells transfected with sh/Ctrl, sh/LINC01559#1, sh/LINC01559#1+empty vector or sh/LINC01559#1+pcDNA3.1/ZEB1. A-B. EdU assay and colony formation assay tested the proliferation ability of GC cells in these four groups. C-D. Flow cytometry analysis and JC-1 experiment detected the apoptotic rate of GC cells in these four groups. E. Transwell assays assessed the migration ability of GC cells in these four groups. F. Western blot experiments detected the protein levels of E-cadherin, N-cadherin, Slug, Twist and ZEB1 in AGS cells of these four groups. G. The quantification bar graphs of western blot bands of Figure 6F and S2F. ^**^P<0.01.
