# Supplementary figures and images for: ZEB1-induced LINC01559 expedites cell proliferation, migration and EMT process in gastric cancer through recruiting IGF2BP2 to stabilize ZEB1 expression
Source: Cell Death Dis. 2021 Apr 6;12(4):349. doi: 10.1038/s41419-021-03571-5 (PMC8024305; doi:10.1038/s41419-021-03571-5)

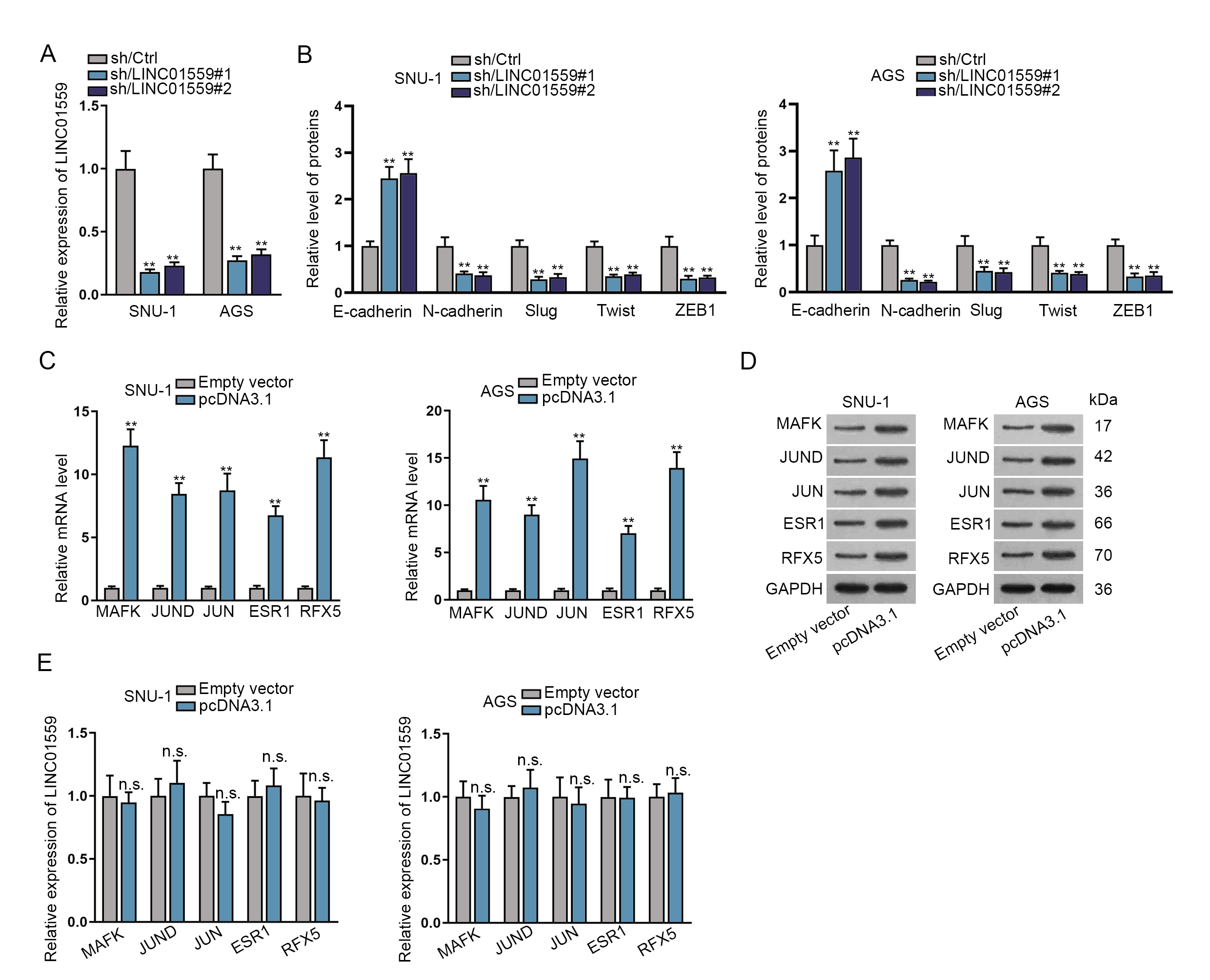

Supplement: Supplementary file 2 — Figure S1 [file 41419_2021_3571_MOESM2_ESM.tif]

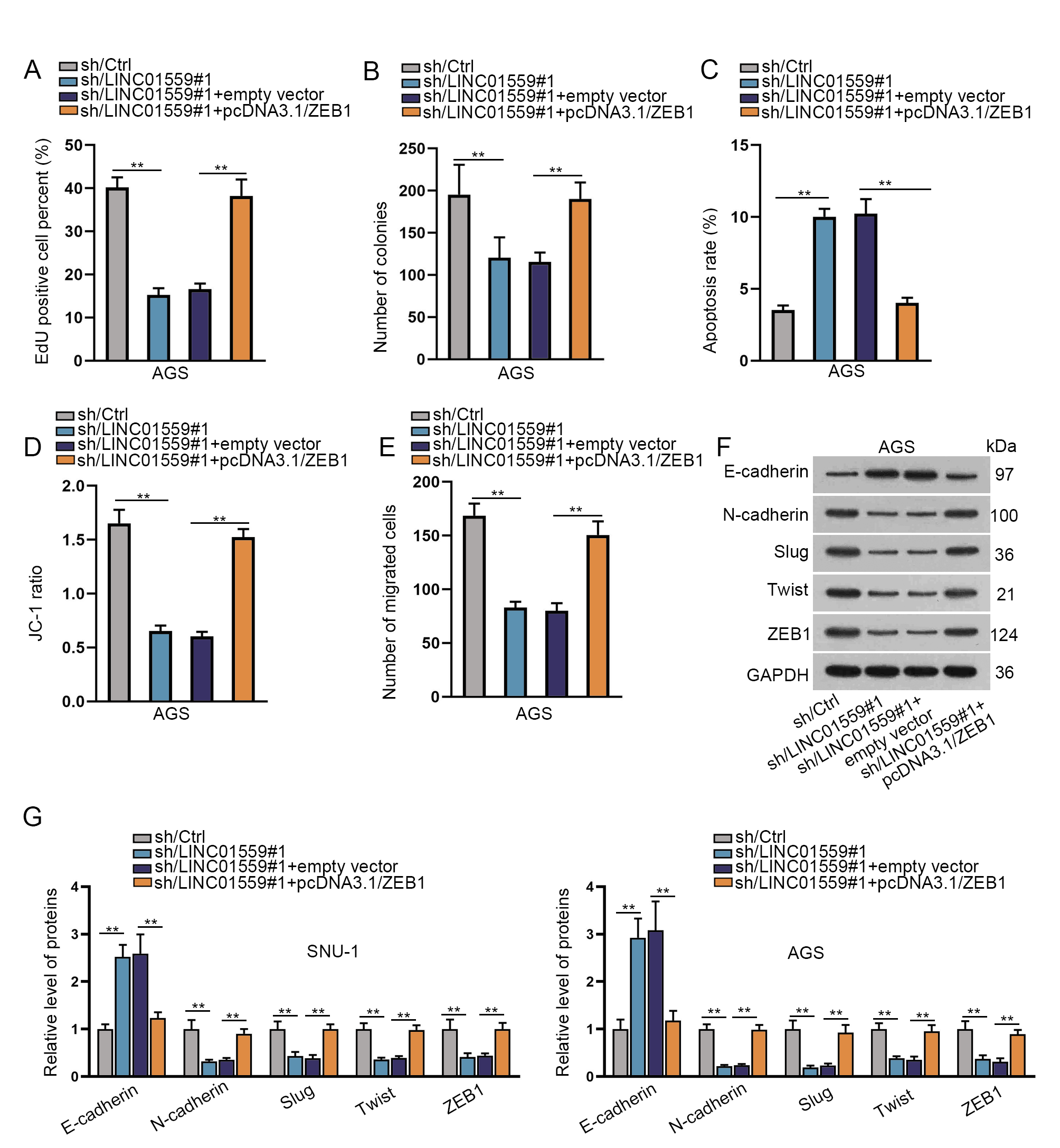

Supplement: Supplementary file 3 — Figure S2 [file 41419_2021_3571_MOESM3_ESM.tif]
